# Progress of Photodynamic Therapy in Gastric Cancer

**DOI:** 10.1155/DTE.5.175

**Published:** 1999

**Authors:** Seishiro Mimura, Hiroyuki Narahara, Toru Otani, Shigeru Okuda

**Affiliations:** Department of Gastroenterology Osaka Medical Center for Cancer and Cardiovascular Diseases 1-3-3, Nakamichi Higashinari-ku Osaka 537-8511 Japan

## Abstract

Progress of photodynamic therapy (PDT) in gastric cancer and the clinical outcome are described in this paper. (1) We included the whole lesion and a 5 mm margin in the field for irradiation. Marking by injection of India-ink showing the irradiation field was performed beforehand. (2) We established the standard light dose to be 90 J/cm^2^ for an argon dye laser and 60 J/cm^2^ for a pulse wave laser. (3) The size of cancerous lesion curable by PDT was expanded from 3 cm in diameter, i.e. 7 cm^2^ in area to 4 cm in diameter, i.e. 13 cm^2^ by employing a new excimer dye laser model, which could emit 4mJ/pulse with 80 Hz pulse frequency. (4) The depth of cancer invasion which could be treated by PDT was increased from about 4 mm, i.e. the superficial part of the submucosal layer (SM-1) to more than 10 mm in depth, i.e. the proper muscular layer. These improvements owe much to the pulse laser, the photodynamic action induced by which permits deeper penetration than that of a continuous wave laser. (5) We employed a side-viewing fiberscope for gastric PDT to irradiate the lesion from an angle of 90°. (6) We designed a simple cut quartz fiber for photoradiation with a spiral spring thickened toward the end. (7) We developed an endoscopic device for photoradiation in PDT which achieves accurate and efficient irradiation. As a result of these improvements a higher cure rate was obtained even with a lower light dose of irradiation.


*Diagnostic and Therapeutic Endoscopy*, Vol. 5, pp. 175–182
Reprints available directly from the publisher
Photocopying permitted by license only

© 1999 OPA (Overseas Publishers Association) N.V.
Published by license under
the Harwood Academic Publishers imprint,
part of The Gordon and Breach Publishing Group.
Printed in Malaysia.

### Progress of Photodynamic Therapy in Gastric Cancer

SEISHIRO MIMURA*, HIROYUKI NARAHARA, TORU OTANI and SHIGERU OKUDA

*Department of Gastroenterology, Osaka Medical Center for Cancer and Cardiovascular Diseases, 1-3-3, Nakamichi, Higashinari-ku, Osaka 537-8511, Japan*

**Progress of photodynamic therapy (PDT) in gastric cancer and the clinical outcome are described in this paper. (1) We included the whole lesion and a 5 mm margin in the field for irradiation. Marking by injection of India-ink showing the irradiation field was performed beforehand. (2) We established the standard light dose to be 90 J/cm$^2$ for an argon dye laser and 60 J/cm$^2$ for a pulse wave laser. (3) The size of cancerous lesion curable by PDT was expanded from 3 cm in diameter, i.e. 7 cm$^2$ in area to 4 cm in diameter, i.e. 13 cm$^2$ by employing a new excimer dye laser model, which could emit 4 mJ/pulse with 80 Hz pulse frequency. (4) The depth of cancer invasion which could be treated by PDT was increased from about 4 mm, i.e. the superficial part of the submucosal layer (SM-1) to more than 10 mm in depth, i.e. the proper muscular layer. These improvements owe much to the pulse laser, the photodynamic action induced by which permits deeper penetration than that of a continuous wave laser. (5) We employed a side-viewing fiberscope for gastric PDT to irradiate the lesion from an angle of 90°. (6) We designed a simple cut quartz fiber for photoradiation with a spiral spring thickened toward the end. (7) We developed an endoscopic device for photoradiation in PDT which achieves accurate and efficient irradiation. As a result of these improvements a higher cure rate was obtained even with a lower light dose of irradiation.**

*Keywords:* Endoscopic treatment, Gastric cancer, Hematoporphyrin derivative (HpD), Photodynamic therapy, Pulse laser

### INTRODUCTION

Since the report on clinical PDT by Dougherty *et al.* [1], and the development of endoscopic PDT for lung cancer by Hayata *et al.* [2], we have studied how to apply this method for gastric cancer since 1979. However, several improvements of techniques and instruments were required to enable PDT for gastric cancer. In Japan, when early gastric cancer is detected, an endoscopic mucosal resection (EMR) is first considered, because the method is simple to carry out and pathologic evaluation of the resected specimen allows assessment of the curativity. Since small mucosal adenocarcinomas without ulceration can be cured with less difficulty by EMR, PDT is indicated in adenocarcinomas with submucosal invasion and/or ulceration. Gastric cancer lesions with ulceration usually show infiltrative growth,

* Corresponding author. Tel.: 81 6.6972 1181. Fax: 81 6 6981 4067.

thus the boundary between the cancerous lesion and surrounding mucosa is frequently unclear. Moreover, the edema of the gastric mucosa induced by laser irradiation further obscures the border. Nevertheless it is essential to overcome these difficulties for localized gastric PDT. PDT is also indicated to patients who would be at high risk if treated by open surgery, e.g. elderly patients and patients with severe organ dysfunction, because it is impossible to cure lymph node metastasis by PDT, and it is also impossible to rule out lymph node micro-metastasis before treatment. In this paper we describe our original ideas and how to put them into practice, in addition to the results in PDT for gastric cancer.

### MATERIALS AND METHODS

### Materials

We performed PDT in 80 patients (84 lesions) with early gastric cancer who were poor risks for conventional surgery. From 1981 to 1990, we treated 38 cases (41 lesions) by PDT with hematoporphyrin derivative [3] (HpD, Photofrin I) or dihematoporphyrin ether/ester (DHE, Photofrin II) and an argon dye laser (ADL: models 171-08 and 375-03, Spectra-Physics, Mountain View, CA, USA). In 1988, we tried a copper vapor dye laser (CuVDL, models HN 102 and HO 102, Niic, Kawasaki, Japan) in PDT. However, since we could not find any advantages of the CuVDL in clinical use in early gastric cancer, 3 cases treated by CuVDL were, therefore, included in the ADL group. From 1990 to 1998, we treated 42 cases (43 lesions) by PDT with polyhematoporphyrin ether/ester (PHE, freeze-dried Photofrin II, Photofrin Injection®) and a pulse laser. The pulse lasers we used were mainly an excimer dye laser [4] (EDL: model EDL PDT-1, Hamamatsu Photonics, Hamamatsu, Japan) and partially between 1995 and 1996 an optical parametric oscillator system pumped by a *Q*-switched Nd:YAG laser [5] (YOL: model iLS-TL-50A, Ishikawajima-Harima Heavy Industries (IHI), Tokyo, Japan). The characteristics of 80 patients (84 lesions) are shown in Table I. The characteristics of lasers we have used for PDT are shown in Table II.

TABLE I Patient characteristics

| Characteristics | Total | ADL | Pulse laser (EDL[a], YOL[b]) |
|---|---|---|---|
| Sex | | | |
| Male | 63 | 26 | 37 |
| Female | 17 | 12 | 5 |
| Age | | | |
| 50–59 (years) | 9 | 4 | 5 |
| 60–69 | 20 | 10 | 10 |
| 70–79 | 36 | 21 | 15 |
| 80–89 | 14 | 3 | 11 |
| 90– | 1 | 0 | 1 |
| Tumor location | | | |
| Upper third | 24 | 17 | 7 |
| Middle third | 29 | 6 | 23 |
| Lower third | 31 | 18 | 13 |
| Histology | | | |
| tub 1[c] | 45 | 22 | 23 |
| tub 2[d] | 30 | 15 | 15 |
| por[e] | 2 | 0 | 2 |
| sig[f] | 7 | 4 | 3 |
| Tumor area | | | |
| −1.0 ($cm^2$) | 17 | 10 | 7 |
| 1.1–2.0 | 8 | 6 | 2 |
| 2.1–4.0 | 30 | 13 | 17 |
| 4.1–7.0 | 21 | 8 | 13 |
| 7.1–12.0 | 6 | 3 | 3 |
| 12.1– | 2 | 1 | 1 |
| Depth of invasion | | | |
| Mucosal | 41 | 22 | 19 |
| Submucosal | 43 | 19 | 24 |
| Gross type | | | |
| Elevated[g] | 13 | 8 | 5 |
| Depressed Ul(−)[h] | 31 | 18 | 13 |
| Depressed Ul(+)[i] | 40 | 15 | 25 |

[a]EDL: excimer dye laser. [b]YOL: optical parametric oscillator system pumped by a *Q*-switched Nd:YAG laser. [c]tub 1: well-differentiated tubular adenocarcinoma. [d]tub 2: moderately differentiated tubular adenocarcinoma. [e]por: poorly differentiated adenocarcinoma. [f]sig: signet-ring cell carcinoma. [g]Elevated: superficial elevated type. [h]Without ulceration. [i]With ulceration.

### Method

The PDT procedure includes three important points, i.e. administration of photosensitizers, laser light irradiation, and pre- and post-procedural management (Table III).

TABLE II Laser characteristics

| Model of laser | Pulse energy (mJ) | Peak power | Pulse width (ns) | Pulse frequency (Hz) | Average output (mw) | Light dose-rates in 1 μs (J) |
|---|---|---|---|---|---|---|
| ADL | — | (300 mW) | — | — | 300 | $3 \times 10^{-7}$ |
| CuVDL | 0.055 | 2.5 kW | 22 | 5,500 | 300 | $5.5 \times 10^{-5}$ |
| EDL, ordinary | 4 | 400 kW | 10 | 40 | 160 | $4 \times 10^{-3}$ |
| EDL, new model | 4 | 400 kW | 10 | 80 | 320 | $4 \times 10^{-3}$ |
| YOL | 5 | 700 kW–1 MW | 5–8 | 50 | 250 | $5 \times 10^{-3}$ |

ADL: argon dye laser. CuVDL: copper vapor dye laser. EDL: excimer dye laser. YOL: optical parametric oscillator system pumped by a *Q*-switched Nd : YAG laser.

TABLE III PDT procedure

1. Intravenous injection of 3 mg/kg of HpD or 2 mg/kg of DHE or PHE.
2. Approximately 50 h later, the entire lesion plus a 5 mm margin of mucosa is irradiated with a laser beam. Standard doses: 90 J/cm$^2$ with ADL, 60 J/cm$^2$ with EDL or YOL.
3. Patients are protected from hypersensitivity to light, and are treated for post-treatment ulcer.

### *Administration of Photosensitizer*

Three mg/kg of HpD or 2 mg/kg of DHE or PHE was given intravenously. The Ministry of Health and Welfare of Japan approved PDT [6] in 1994, and Photofrin Injection® became commercially available in 1995.

### *Laser Light Irradiation*

Approximately 50 h after intravenous injection of photosensitizer, the entire lesion plus a 5 mm wider perimeter of mucosa was irradiated with a laser beam at 630 nm wavelength transmitted endoscopically. The laser beam was condensed and transmitted through a 400-μm core diameter quartz fiber via the working channel. For extensive lesions, the irradiation field was first set on the distal portion of the lesion, and after delivering the scheduled dose of light there, the field of irradiation was shifted to the remaining part to irradiate the entire region as uniformly as possible. Assuming that the irradiation was uniform, the total energy intensity (J/cm$^2$) was calculated by the following formula:

$$\begin{aligned}\text{total energy intensity (J/cm}^2) &= \text{average output (W)} \times \text{time (s)} \\ &\quad /\text{total irradiated area (cm}^2) \\ &\quad \text{(in a continuous wave laser),}\end{aligned}$$

or

$$\begin{aligned}&= \text{pulse energy (J/pulse)} \\ &\quad \times \text{pulse frequency (pulse/s)} \\ &\quad \times \text{time (s)/total irradiated area (cm}^2) \\ &\quad \text{(in a pulse laser).}\end{aligned}$$

*Side-viewing fiberscope* In gastric PDT we usually used a side-viewing gastro-fiberscope (model GF type B3, OES GF 10 and 20, Olympus, Tokyo, Japan) to observe the lesion from an angle of 90°, keeping a certain distance between the fiber tip for irradiation and the lesion. In this way accurate photoradiation is possible throughout the stomach.

*New fiber for photoradiation* When using the side-viewing fiberscope, we need to bend the quartz fiber 90° with a curvature radius of 20 mm. Although the 400-μm core diameter quartz fiber did not mechanically break, when bent in such a fashion it would form an "elbow", and the laser beam would ooze from the core at the elbow and overheat. Currently, we therefore developed a new fiber with a spiral spring [7] (Hamamatsu Photonics, Hamamatsu, Japan). The spring, which was made of a stainless steel wire 100 μm in diameter, was made 6 mm in length, thickened toward the end. It was fixed at the fiber tip and encased in a 1.5 mm outer diameter Teflon tube. It did not disturb passage of air but protected the fiber from destruction, because it ensured an even curvature and prevented the formation of an "elbow". This fiber is low-cost, can withstand bending and moreover is easy to repair.

*A new device for PDT* In gastric PDT there was the problem of the dazzling reflection of the red laser light during photoradiation. Initially we placed a green filter (models SP-15 and SP-19, Fuji Filter, Tokyo, Japan) on the eyepiece of the fiberscope and in front of the charge coupled device (CCD) of an old OES-TV system (model OTV-F2, Olympus). The red spot of laser light could be observed clearly, but the mucosal image turned to green and dark, and the boundary of the cancerous lesion became unclear. Recently, we therefore developed a new device [8] for photoradiation which consisted of a conventional fiberscope and a new OES-TV system (model OTV-S5, Olympus). In front of the CCD we inserted a new interference filter which had specific absorption of red light with a 2.3% transmissivity at 630 nm wavelength and a 50 nm absorption band of full width at half maximum, while the average transmittance in the visible region was 90% (Hamamatsu Photonics). In this way we could observe the mucosa clearly during the entire course of irradiation.

### *Pre- and Post-Procedural Management*

Soon after injection of the photosensitizer, the patients were placed in a dark room. After PDT, the patients were kept in bed without oral feeding and were given continuous intravenous glucose and saline with an H2-blocker and antibiotics for two days to allow healing of ulcers. During this period, the patients were kept in dim light and their faces and hands were coated with antisunburn cream. These restrictions were gradually reduced, and the patients returned to normal conditions after one week.

### Evaluation of Response

The response was evaluated by endoscopy with biopsy and cytology in follow-up examinations. They were followed up by endoscopy 2, 3 and 6 months later, then every 6 months for 2 years, and thereafter once a year. To detect metastasis to the lungs, liver, and abdominal lymph nodes, the patients were also followed up by chest X-ray and ultrasonic echography. Curativity was divided into two categories: 'cure' or 'no cure'. Although it is impossible to prove that cancer was completely cured based on clinical examinations, our definition of 'cure' was when neither local recurrence nor metastasis were detected by follow-up examinations at 1 year. Cases classified as partial response and recurrence after 1 year, therefore, were classified as 'no cure' in our evaluation [9].

### RESULTS

### Patient Characteristics

Patient characteristics and response in relation to the kind of laser used are summarized in Table IV. The effects of PDT with the ADL in early gastric cancer lesions, according to the depth of cancer invasion are shown in Table V. The rate of cure for mucosal carcinomas was 59% (13/22), that of submucosal carcinomas was 53% (10/19) and the overall rate was 56% (23/41). The effects of PDT with the pulse laser, that is EDL or YOL, according to the depth of cancer invasion are shown in Table VI. The pulse laser improved cure rates markedly to 95%, i.e. 18 of 19 mucosal cancers, and 75%, i.e. 18 of 24 in submucosal cancers, and 84%, i.e. 36 of 43 overall.

Among them the most extensive lesions cured by PDT were 19 and 12 $cm^2$ lesions of the mucosal and submucosal carcinoma, respectively. Cure was obtained in a 79-year-old man with a lesion spreading 19 $cm^2$ in the mucosal layer. The superficial elevated type lesion was located in the middle third of the stomach on the lesser curvature, and biopsy revealed well-differentiated tubular adenocarcinoma. Photoradiation with the YOL through a quartz fiber with a concave lens (Ishikawajima-Harima Heavy Industries) was performed at 53 h after administration of PHE, giving 1,080 J over

TABLE IV Response in relation to treatment with ADL or pulse laser

| Kind of laser | Argon dye laser | | | Pulse laser (EDL[a], YOL[b]) | | |
|---|---|---|---|---|---|---|
| Characteristics | Total | CR[c] | Non-CR | Total | CR | Non-CR |
| Sex | | | | | | |
| Male | 26 | 12 | 14 | 37 | 31 | 6 |
| Female | 12 | 8 | 4 | 5 | 4 | 1 |
| Age | | | | | | |
| 50–59 (years) | 4 | 2 | 2 | 5 | 5 | 0 |
| 60–69 | 10 | 3 | 7 | 10 | 7 | 3 |
| 70–79 | 21 | 14 | 7 | 15 | 14 | 1 |
| 80–89 | 3 | 1 | 2 | 11 | 8 | 3 |
| 90– | 0 | 0 | 0 | 1 | 1 | 0 |
| Tumor location | | | | | | |
| Upper third | 17 | 11 | 6 | 7 | 7 | 0 |
| Middle third | 6 | 3 | 3 | 23 | 18 | 5 |
| Lower third | 18 | 9 | 9 | 13 | 11 | 2 |
| Histology | | | | | | |
| tub 1[d] | 22 | 11 | 11 | 23 | 21 | 2 |
| tub 2[e] | 15 | 10 | 5 | 15 | 12 | 3 |
| por[f] | 0 | 0 | 0 | 2 | 2 | 0 |
| sig[g] | 4 | 2 | 2 | 3 | 1 | 2 |
| Tumor area | | | | | | |
| −1.0 ($cm^2$) | 10 | 7 | 3 | 7 | 7 | 0 |
| 1.1–2.0 | 6 | 5 | 1 | 2 | 2 | 0 |
| 2.1–4.0 | 13 | 8 | 5 | 17 | 12 | 5 |
| 4.1–7.0 | 8 | 2 | 6 | 13 | 11 | 2 |
| 7.1–12.0 | 3 | 1 | 2 | 3 | 3 | 0 |
| 12.1– | 1 | 0 | 1 | 1 | 1 | 0 |
| Depth of invasion | | | | | | |
| Mucosal | 22 | 13 | 9 | 19 | 18 | 1 |
| Submucosal | 19 | 10 | 9 | 24 | 18 | 6 |
| Gross type | | | | | | |
| Elevated[h] | 8 | 4 | 4 | 5 | 4 | 1 |
| Depressed Ul(−)[i] | 18 | 10 | 8 | 13 | 12 | 1 |
| Depressed Ul(+)[j] | 15 | 9 | 6 | 25 | 20 | 5 |

[a]EDL: excimer dye laser. [b]YOL: optical parametric oscillator system pumped by a *Q*-switched Nd : YAG laser. [c]CR: complete response. [d]tub 1: well-differentiated tubular adenocarcinoma. [e]tub 2: moderately differentiated tubular adenocarcinoma. [f]por: poorly differentiated adenocarcinoma. [g]sig: signet-ring cell carcinoma. [h]Elevated: superficial elevated type. [i]Without ulceration. [j]With ulceration.

TABLE V Rates of cure of early gastric cancer obtained by PDT with an ADL[a] according to depth

| Depth of invasion | Number of lesions | Number of CR lesions (%) |
|---|---|---|
| Mucosa | 22 | 13 (59%) |
| Submucosa | 19 | 10 (53%) |
| Total | 41 | 23 (56%) |

[a]ADL: argon dye laser.

TABLE VI Rates of cure of early gastric cancer obtained by PDT with pulse laser (EDL[a], YOL[b]) according to depth

| Depth of invasion | Number of lesions | Number of CR lesions (%) |
|---|---|---|
| Mucosa | 19 | 18 (95) |
| Submucosa | 24 | 18 (75) |
| Total | 43 | 36 (84) |

[a]EDL: excimer dye laser. [b]YOL: optical parametric oscillator system pumped by a *Q*-switched Nd : YAG laser.

$27\,cm^2$, which was calculated as $40\,J/cm^2$ total energy intensity.

Cure was also achieved by PDT in a 64-year-old man with an extensive early gastric cancer lesion with submucosal invasion. The lesion was the superficial depressed type with ulceration, located in the middle third of the stomach on the lesser curvature, spreading over $12\,cm^2$. Biopsy revealed signet-ring cell carcinoma and the depth of invasion suggested by endoscopic ultrasonography was the full depth of the submucosal layer (SM-3) bordering on the proper muscular layer. PDT was carried out with an EDL using a simple cutting quartz fiber without lens or diffuser, giving $48\,J/cm^2$ to a $15\,cm^2$ area [10].

During the same periods, we treated 8 patients with advanced gastric cancer by PDT, 2 with an ADL and 6 with an EDL and one of the latter achieved a complete cure, that is complete remission without recurrence at 1 year.

### Side Effects

In one case treated with the EDL, massive bleeding occurred from an ulcer produced after PDT. The hemoglobin decreased to 6.2 g/dl, but recovered following conservative treatment such as blood transfusion, so endoscopic hemostasis did not have to be carried out. In four other cases, mild cutaneous reaction and photosensitivity lasted several weeks. In one other case, mild cutaneous pigmentation lasted several months. The main abnormality revealed by laboratory tests was decrease of total protein, which was observed in 32 patients (40%) and was thought to be caused by protein loss from the base of the ulcer that developed after PDT and acute gastritis surrounding the ulcer. Fourteen patients out of 32 recovered within several months with a normal diet, while 18 patients, with a tendency toward hypoalbuminemia before treatment, such as in liver cirrhosis, required intravenous injections of 150–1100 ml of 25% human albumin. Slight liver dysfunction was seen in 5 patients, lasting for several weeks. No serious adverse reactions were seen.

### DISCUSSION

### The Irradiation Field

It was our original intention to include a normal mucosal margin of 5 mm width for irradiation, and this was carried out, with only few exceptions, since the first treated case. For extensive lesions, the irradiation field was first set on the distal portion of the lesion, and after delivering the scheduled dose of light there, the field of irradiation was shifted to the remaining part. We began at the distal portion because the edema of the mucosa induced by irradiation will conveniently raise the lesion toward a proximal direction, and also because photoradiation can cause the gastric wall to become spastic and would push out the fiberscope gradually, therefore it would become difficult to insert the fiberscope into the distal portion in the later period of the treatment.

### Energy Intensity

The appropriate energy intensity level is also important. Kato *et al.* [11] endoscopically delivered more than $200\,J/cm^2$ by an ADL and more than $100\,J/cm^2$ by an EDL for lung cancer. Udagawa [5] reported the results of clinical trials of PDT in several organs using a YOL, in which he reported $100–300\,J/cm^2$ for early lung cancer and CIS of the uterine cervix, and $60–180\,J/cm^2$ for early gastric cancer and superficial esophageal cancer. We gave $204\,J/cm^2$ for our first case of superficial esophageal cancer and $90\,J/cm^2$ for our first case of early gastric cancer. After trials using several doses, we recognized that the tumor response did not correspond to the light dose, while homogeneous irradiation including a sufficient margin was essential. Thereafter, we established the standard light dose to be $90\,J/cm^2$ for the ADL and $60\,J/cm^2$ for the EDL and YOL for superficial esophageal cancer and early gastric cancer. There has been much discussion why smaller doses with the pulse laser are enough. In spite of no clinical randomized comparative trials between a continuous wave laser and a pulse laser, it was proved that $60\,J/cm^2$ of the EDL or YOL

was enough to obtain a complete response (CR) for superficial esophageal cancer and early gastric cancer [12–15].

### PDT for Extensive Lesions

Although the size of the lesion which can be treated by PDT is limited by the duration of treatment time tolerated by the patients, i.e. within approximately 1 h in our experience, even if a patient can bear an additional irradiation session, the lesion and the surrounding mucosa will have become edematous with partially hemorragic necrosis, and moreover the surface will be covered with a white, partially black membrane which consists of a coagulated mixture of mucus, serum protein exudate and blood. It is difficult to remove the membrane without causing bleeding. The maximum size of a lesion that can be treated by PDT is the maximum area including the whole lesion plus a 5 mm margin to which the laser can give the standard dose of 60 $J/cm^2$ in 1 h. This depends on the average output of the laser. In the new EDL model as shown in Table II, the pulse frequency can be increased to 80 Hz, yielding an average output of 320 mW. By shortening the irradiation time for a certain energy, i.e. 60 J, the application of PDT can be extended to larger lesions up to 4 cm in diameter (13 $cm^2$) giving treatment for 1 h [16]. In practice, extensive lesions spreading over 19 $cm^2$ in the mucosa and 12 $cm^2$ in the submucosa were cured by irradiation with only 40 and 48 $J/cm^2$, respectively as described above. These results suggest that PDT can be indicated for more extensive lesion with less energy intensity.

### Depth of Cancer Invasion

The depth of photodynamic action depends on three factors: the transmissivity of the tissue, the density and sensitivity of photosensitizer, and the laser characteristics including its wavelength. Does the depth of photodynamic action also depend on the laser characteristics apart from its wavelength? In spite of using the same energy intensity, it was demonstrated not only in animal tumors [17] but also in clinical cases [18] that a pulse laser with an extremely high peak power had more efficient photodynamic action than a continuous wave laser such as an ADL or a high frequency pulse laser such as a CuVDL. However, the theory proving why a pulse laser could be superior to a continuous wave laser was not developed until 1994, when Takemura *et al.* [19] proved it by introducing the concept of light dose-rate effects. He proposed that if there are thresholds of dose-rates of laser light to activate the photosensitizer, the depth would vary according to the light dose-rates. The hypothesis of thresholds of light dose-rates was subsequently recognized. In normal PDT the intensity of the laser light on the surface of the lesion is enough to activate the photosensitizer, whereas the deeper the light penetrates the tissue the weaker it would become. Finally, the intensity of the light would weaken to the threshold of the light dose-rates. This point corresponds to the maximum depth of photodynamic action. As the light dose-rates, i.e. the energy intensity for 1 s, depends on the average output, it might appear that there would be no difference between a continuous wave laser and a pulse laser. However, it is necessary to give strict consideration to the consumption time period for activation of the photosensitizer and production of singlet oxygen, which is approximately 1 μs ($10^{-6}$). Therefore, if energy greater than the threshold can be given in 1 μs or less will decide the effect of the photodynamic action. The energy intensity in 1 μs of an ADL of 300 mW irradiated to an area of 1 $cm^2$ is $3 \times 10^{-7}$ J, whereas that of an EDL or YOL is $4 \times 10^{-3}$ and $5 \times 10^{-3}$ J, respectively, that is more than 10,000 times that of the ADL. The light dose-rates for 1 μs for each laser are shown in Table II. Although it is not yet recognized internationally that the energy intensity for 1 μs is the most important factor to decide the depth of effective photodynamic action, the therapeutic effects on gastric cancer more than 10 mm in depth supports this hypothesis.

In 1993, we eventually succeeded in treating an 80-year-old man with advanced gastric cancer by

PDT [20]. This lesion was advanced gastric cancer type 2, localized depressed type, located in the middle third of stomach and its area was 13 $cm^2$. Biopsy revealed a moderately differentiated tubular adenocarcinoma (tub 2), and the depth of invasion suggested by endoscopic ultrasonography was PM, i.e. the cancer invaded the proper muscular layer, revealing 10 mm in tumor thickness. PDT was performed with PHE and a new EDL using a quartz fiber with a concave lens, delivering 48 $J/cm^2$ to an area of 15 $cm^2$, and complete cure was obtained. Follow up for 1 year after PDT demonstrated no local recurrence of cancer and absence of metastasis was indicated by chest X-ray, ultrasonic echography and computed tomography.

### CONCLUSION

Through improvements of techniques and instruments a high rate of cure can be obtained in submucosal gastric cancer including lesions with ulceration by PDT with PHE and a pulse laser. The new EDL model which can emit 80 Hz pulse frequency allows more extensive lesions to be treated. Finally, a complete remission without recurrence was achieved by PDT in one case of advanced gastric cancer.

### *References*

[1] Dougherty, T.J., Lawrence, G., Kaufman, J.H. *et al.* Photoradiation in the treatment of recurrent breast carcinoma. *J. Natl. Cancer Inst.* 1979; **62**: 231–237.

[2] Hayata, Y., Kato, H., Konaka, C. *et al.* Hematoporphyrin derivative and laser photoradiation in the treatment of lung cancer. *Chest* 1982; **81**: 269–277.

[3] Lipson, R.L. and Baldes, E.J. The photodynamic properties of a particular hematoporphyrin derivative. *Arch. Dermatol.* 1960; **82**: 508–516.

[4] Hirano, T., Ishizuka, M., Suzuki, K. *et al.* Photodynamic cancer diagnosis and treatment system consisting of pulsed lasers and an endoscopic spectro-image analyzer. *Lasers Life Sci.* 1989; **3**: 99–116.

[5] Udagawa, T., Takaoka, K., Inoue, K. *et al.* "Development of YAG-OPO laser for PDT application", in *7th Japanese Chapter Anniversary Meeting of International Photodynamic Association* (*JCIPA*), Nishisaka, T. (Ed.) *Proc. Recent Progress of Photodynamic Therapy*, 1997: 77–80.

[6] Hayata, Y., Kato, H., Huruse, K. *et al.* Photodynamic therapy of 168 early stage cancers of the lung and oesophagus: a Japanese multi-centre study. *Lasers Medi. Sci.* 1996; **11**: 255–259.

[7] Mimura, S., Narahara, H., Otani, T. *et al.* "Development of a fiber for endoscopic photoradiation in photodynamic therapy", in *8th Japanese Chapter Anniversary Meeting of International Photodynamic Association* (*JCIPA*) *at Osaka*, 1998.

[8] Mimura, S., Narahara, H., Otani, T. *et al.* "Development of a device for endoscopic photoradiation in photodynamic therapy", in *7th International Photodynamic Association Biennial Meeting at Nantes*, 1998.

[9] Mimura, S., Ichii, M., Imanishi, K. *et al.* Evaluation of photodynamic therapy. *Dig. Endosc.* 1990; **2**: 265–274.

[10] Mimura, S., Narahara, H., Otani, T. *et al.* "Photodynamic therapy for early gastric cancer: its application for wider lesions," in *5th International Photodynamic Association Biennial Meeting*, Denis A. Cortese (Ed.) *Proc. SPIE* 1995; **2371**: 522–525.

[11] Kato, H., Horai, T., Furuse, K. *et al.* Photodynamic therapy for cancers: a clinical trial of porfimer sodium in Japan. *Jpn. J. Cancer Res.* 1993; **84**: 1209–1214.

[12] Yoshida, K., Suzuki, S., Mimura, S. *et al.* Photodynamic therapy for superficial esophageal cancer: a phase III study using PHE and excimer dye laser. *Jpn. J. Cancer Chemother.* 1993; **20**: 2063–2066.

[13] Mimura, S., Ito, Y., Nagayo, T. *et al.* Cooperative clinical trial of photodynamic therapy with Photofrin II and excimer dye laser for early gastric cancer. *Lasers Surg. Medi.* 1996; **19**: 168–172.

[14] Yoshida, K., Suzuki, S., Mimura, S. *et al.* A clinical study of photodynamic therapy for superficial esophageal carcinoma by YAG-OPO laser. *Diag. Ther. Endosc.* 1998; **4**: 173–176.

[15] Mimura, S., Narahara, H., Hirashima, T. *et al.* Cooperative clinical trial of photodynamic therapy for early gastric cancer with Photofrin Injection® and YAG-OPO laser. *Diag. Ther. Endosc.* 1998; **4**: 165–171.

[16] Narahara, H., Mimura, S., Otani, T. *et al.* "The application of photodynamic therapy for gastric cancer using Photofrin II and an excimer dye laser," in *6th International Photodynamic Association Biennial Meeting at Melbourne*, 1996.

[17] Okunaka, T., Kato, H., Konaka, C. *et al.* A comparison between argon-dye and excimer-dye laser for photodynamic effect in transplanted mouse tumor. *Jpn. J. Cancer Res.* 1992; **83**: 226–231.

[18] Mimura, S., Ichii, M. and Okuda, S. Photodynamic therapy for early gastric cancer using excimer dye laser. *Photodynamic Therapy and Biomedical Lasers*, Spinelli, P., Dal Fante, M. and Marchesini, R. (Eds.) pp. 272–276, Elsevier Science Publishers B.V., Amsterdam 1992.

[19] Takemura, T., Umeuchi, S., Nakajima, S. *et al.* "Mechanism of photodynamic therapy (PDT): investigation of sensitizer dose and light dose-rate effects," in *5th International Photodynamic Association Biennial Meeting*, Denis A. Cortese (Ed.) *Proc. SPIE* 1995; **2371**: 351–354.

[20] Mimura, S., Narahara, H., Otani, T. *et al.* "A case report of advanced gastric cancer treated by photodynamic therapy using Photofrin II and an excimer dye laser," in *6th International Photodynamic Association Biennial Meeting at Melbourne*, 1996.

